# The Prevalence of Virulence Genes and Virulotypes of *Escherichia coli* Strains Isolated from Hospital Wastewaters in Tehran, Iran

**Published:** 2018-05

**Authors:** Reza RANJBAR, Omid FARAHANI

**Affiliations:** 1. Molecular Biology Research Center, Systems Biology and Poisonings Institute, Baqiyatallah University of Medical Sciences, Tehran, Iran; 2. Dept. of Microbiology, Islamic Azad University, Varamin-Pishva Branch, Tehran, Iran

**Keywords:** *Escherichia coli*, Hospital wastewater, Virulence factors

## Abstract

**Background:**

Due to the widespread different pathogenic strains, *Escherichia coli* lead many severe to normal diseases worldwide. Finding the relation of clones with genomic content and clinical features is a key point to recognize the high potential-invasive strains. Specific virulence factors include adhesions, invasions, toxins, and capsule are the main determinants of pathogenic factors of *E. coli* strains.

**Methods:**

From Jun 2014 to Jun 2016, *E. coli* isolates recovered using standard bacteriological methods from wastewater sources in different hospitals in Tehran, Iran, were monitored to recognize the virulence genes by polymerase chain reaction (PCR) assay.

**Results:**

The high and low presences of virulence factors were *fimH, 76% and afa*, *13%,* respectively.

**Conclusion:**

The results indicated the potential pathogenicity of *E. coli* strains circulating in hospital wastewaters in Tehran, Iran.

## Introduction

As one of the main sources of risk issues related to public health, there is an increasing concern about water contaminations despite recent development in the field of water treatment technologies (1). Coastal zones with the high percentage of untreated sewage injected into waters play a significant role in terms of pollution formation supposed to be the main cause for the greatest number of public health hazard worldwide (2–4) containing waterborne severe diseases and infections like typhoid fever, cholera, dysentery and traveller’s diarrhea, caused by different types of bacterial pathogens (5). Expanding the spectrum of waterborne infections (6), lack of regular monitoring of waterborne pathogens, as well as accurate and cost-effective diagnostic tests, are the major issues required to be concerned in order to protect public health, prevent and control the infections transmitted by waterborne pathogens. The presence of *E. coli* in a variety of infections (7–9), and its diverse clinical spectrum ranging from asymptomatic bacteriuria (reported to have a prevalence of 4%–6% in young women and up to 20% in elderly women (10, 11)) to acute cystitis to pyelonephritis is considered as one of the most common troublesome medicinal issue in an extensive variety of countries all around the world. The complex pathogenesis analyses are infected by both the host biological and behavioral, and the virulence characteristics of the infecting uropathogenic (12). Its propensity to rehash and the certifiable test posed to practical chemotherapy make it worth focus on the virulence repertoire of *E. coli* strains isolated from infected samples.

The range of well-recognized virulence factors (VF), for instance, fimbrial and afimbrial adhe-sins, toxins and siderophores and new potential ones have been implicated to *E. coli* strains to agitate the host prosperity congruity (13). Many genes are involved, which means that interaction of various virulence genes with host immune system resulted in infection. The exact role of most virulence determinants, however, is still not clearly known in many infection pathogenesis (14).

Conventional methods for detection of *E. coli* based on culturing were time-consuming and not enough efficient in order to identification of pathogenic strains. Improvement of molecular technology has conducted apprehensive studies in the field of uropathogenic *E. coli* (8, 12, 15–24). The convenient and rapid approach of polymerase chain reaction (PCR) leads to figure out an outstanding assessment of virulence determinants detected which may be useful for diagnosis applications and therapeutic strategies (25–28). Unfortunately, despite the importance of uropathogenic *E. coli*, few studies have been performed for monitoring of uropathogenic *E. coli* especially in waters and wastewaters samples (29).

Recent studies are carried out interested to assess the prevalence of different operons coding for virulence factors among *E. coli* strains isolated from various samples from different sources and establish the data bank containing the molecular characteristics of *E. coli* strains to circulate in different countries.

In the present study, *E coli* isolates from wastewater sources of different hospitals in Tehran, Iran, were monitored to recognize the virulence genes by polymerase chain reaction (PCR) assay.

## Materials and Methods

### Sample collection and processing

*E. coli* strains isolated from hospital wastewater sources over a 24-months sampling period (Jun 2014–Jun 2016) were included in the study. For further analysis, sampling bottles were shipped to the Laboratory in Molecular Biology Research Center, Baqiyatallah, Tehran, Iran. Standard Methods for the Examination of Water and Wastewater 22^nd^ edition were used as the basic methods for culture-based tests (30). All samples were analyzed for the presence of *E. coli*. *E. coli* colonies from agar plates were picked and streaked for purity on EMB agar. Well-isolated colonies of purified *E. coli* were stored for long-term storage in SKIM Milk media with 20% (v/v) glycerol and at −20 colThe *E. coli* strains were isolated by minimal standard bacteriological test and identified compared with the *E. coli* laboratory reference using conventional biochemical tests.

### PCR

*E. coli* isolates were tested for the presence of specific primers used to amplify sequences of the type 1 fimbriae (*fimH*), pili associated with pyelonephritis (*pap*), S fimbriae (*sfa*), afimbrial adhesins (*afa*), hemolysin (*hly*), and cytotoxic necrotizing factor (*cnf*), aerobactin (*aer*). Details of primer sequences, predicted sizes of the amplified products and specific annealing temperatures are given in Table 1.

**Table 1:** Characteristics of primer sequences used for PCR assays

***Virulence factor***	***Target gene(s)***	***Primer sequence (5′-3′)***	***Size fragment (bp)***
Tape 1 fimbriae	*fimH*	F:GAGAAGAGGTTTGATTTAACTTATTG R:AGAGCCGCTGTAGAACTGAGG	559
P fimbriae	*pap*	F:GACGGCTGTACTGCAGGG TGTGGCG R:ATATCCTTTCTGCAGGGATGCAATA	328
Afa adhesins	*afa*	F:GCTGGGCAGCAAACTGATAAC TCTC R:CATCAAGCTGTTTGTTCGTCCGCCG	750
Haemolysin	*hlyA*	F:AACAAGGATAAGCACTGTTCTGGCT R:ACCATATAAGCGGTCATTCCCGTCA	1177
Cytotoxic necrotizing factor	*cnf*	F:TTATATAGTCGTCAAGATGGA R:CACTAAGCTTTACAATATTGA	639
Aerobactin	*aer*	F:TACCGGATTGTCATATGCAGACCGT R:AATATCTTCCTCCAGTCCGGAGAAG	602

Detection of sequences of target genes was done by PCR (31). PCR amplification of bacterial DNA extracts was performed in 20 μl of template DNA (1 μl), 10 pmoles of each primer, the four deoxynucleoside triphosphates (each at 10 μM) and 1.25 U Taq DNA polymerase in 2x PCR buffer containing MgCl_2_. For finding the optimum amplification procedure, the denaturation and annealing conditions were described in Table 2. The final reaction mixture (6μl) underwent gel electrophoresis in 1.5% agarose containing in 100 ml TBE (1X) buffer (1,6 M Tris-EDTA, 0,02 M boric acid) per ml. Amplified DNA fragments of specific sizes were detected by UV induced fluorescence. Overall, 1000 bp DNA ladder (Viogene) was used for determining the sizes of the amplicons.

**Table 2:** PCR conditions of target genes

***Genes***	***Initial denaturation (°C/min)***	***Denaturation (°C/s)***	***Annealing (°C/s)***	***Extension (°C/s)***	***Final extension (°C/min)***
*fimH*	95/5	94/60	59/60	72/60	72/7
*pap*	95/5	94/60	63/60	72/60	72/7
*afa*	95/5	94/60	65/60	72/60	72/7
*hlyA*	95/5	94/60	62/60	72/60	72/7
*cnf*	95/5	94/60	57/60	72/60	72/7
*aer*	95/5	94/60	68/60	72/60	72/7

**Fig. 1:**
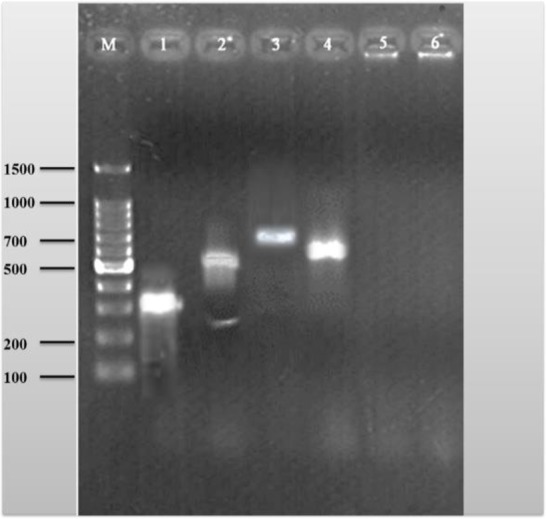
Detection of *fimH* (559 bp), *aer* (602 bp), *pap* (328 bp), and *afa* (750 bp) gene by PCR in hospital wastewaters. Lane 5 and 6: negative representative *hlyA* and *cnf* genes

The amplified DNA product was visualized by standard submarine gel electrophoresis using 10 μL of the final reaction mixture on a 2% agarose gel. Amplified DNA fragments of specific sizes were located by UV fluorescence, after staining with ethidium bromide. The 100-bp ladder was used as a standard for determining molecular size of PCR products (Fig. 1).

## Results

Eighty strains of *E. coli* were isolated from hospital wastewater sources and subjected to PCR assay in order to determine the prevalence of virulence genes.

The frequencies and profiles of virulence genes are shown in Table 3 and 4, respectively. The prevalence of genes of fimbrial adhesive systems was 76% and 16% for *fimH*, and *pap,* respectively. *Afa* afimbrial adhesins were detected in 13% of strains. The prevalence of 53% was identified for the *aer* gene. The presence of two genes encoding toxins, i.e. *hly* and *cnf* were not detected. Sixty-one strains shared DNA sequences related to *fimH* and other genes associated as *aer*, *pap*, and *afa. FimH* adhesin was the most prevalent virulence factor detected, having occurred in 76% of strains, and it was detected in 16% of operon *pap*. The studied strains of the total *fimH* showed adhesin-encoding operon *fimH* (95%), *fimH-afa* (20%), *fimH-afa-pap* (2.5%) and *fimH-pap* (1.2%) and 51 of them showed the other VF gene: *Aer- fimH* (47%), *Aer- fimH-afa* (6.2%) and *Aer- fimH-pap* (10%) (Table 4). The high presence of adhesion genes and the low presence of genes encoding toxins were illustrated in our finding.

**Table 3:** Frequency of virulence genes among 80 *E. coli* isolates

***Virulence gene***	***Total (n = 80), n (%)***
*fimH*	61(76)
*aer*	43(53)
*Pap*	13(16)
*afa*	11(13)
*hlyA*	0 (0)
*cnf*	0 (0)

**Table 4:** Nine different patterns for virulence gene profiles

***Profile names***	***Virulence gene patterns***	***Total (n = 80), n (%)***
[Type 1]	*fimH-afa*	16 (20)
[Type 2]	*fimH-afa-pap*	2 (2.5)
[Type 3]	*afa-pap*	2(2.5)
[Type 4]	*fimH-pap*	1(1.2)
[Type 5]	*Aer- fimH*	38(47)
[Type 6]	*Aer- fimH-afa*	5(6.2)
[Type 7]	*Aer- fimH-pap*	8(10)
[Type 8]	*Aer- afa*	5(6.2)
[Type 9]	*Aer-pap*	8(10)

## Discussion

Finding the relation of genetic virulence traits of the clinical isolates and the type of infections will influence the treatment of these infections. Adhesins, toxins and iron-chelating factors (siderophores) were reported as the main virulence factors (VFs) of *E. coli* (32) while afimbrial adhesins (*afa*), pfimbrial adhesins (*pap*) and s-fimbrial adhesins (*sfa*) were indicated as those adhesins by which UPEC typically attach to epithelial cells lining (33).

Intimin, as one of the pathogenic factor, was playing an important role in virulence helping bacteria to attach the epithelial cell lining (28, 34). A recent research about cytotoxic necrotizing and haemolytic factors was conducted (18). Forty percent of UPEC produce cytotoxic necrotizing factor 1(*cnf1*) involved in the dissemination and persistence of cells in the urinary tract. Aerobactin, the iron chelating agent, encoded by *aer* have been reported as a determinate factor to enabling colonization of *E. coli* in iron-poor environments (35). Promotion of bacterial invasion through the epithelial barrier is considered due to the cytolytic effect of α-hemolysin (*hlyA*) encoded by α-*hly*. In this study, the high percentage of adhesins gene could be related to the pathogenicity of the isolated strains as adherence is the most important pathogenicity determinant (36). *fimH* was highly conserved in UTI isolates, which confirms its crucial role during colonization of the urinary tract (36–39). The presence of *pap* gene had been considered in association with pyelonephritis; therefore, lower percentages of *pap* gene (16%) may propose the lower capabilities of isolated strains to colonize kidneys and generate pyelonephritis (40, 41). The *hlyA* and *cnf* genes indicated the positive relationship with pathogenicity island PAI II_J96_, and the pathogenicity island PAI I_CFT073_ is related to *aer* gene as well as with *hlyA* and *pap* operon (42–46).

Molecular methods particularly DNA-based detection techniques help us help us in the field of diagnostic and molecular epidemiology of pathogenic microbial strains (47–48). The technique used in current study was able of to elucidate successfully the pathogenicity status of the strains under the study.

## Conclusion

Consequently, we represent the high frequency of *fimH* gene among isolated strains of *E. coli* from wastewater samples followed by new patterns of frequencies in association with virulence genes of *aer, pap, afa,* and *fimH*. PCR typing acts as a useful method to determine the virulence gene profiles of *E. coli* strains and detect the complete data from genome content in association with different *E. coli* strains that may cause severe illnesses worldwide. The high percent of virulence genes of *E. coli* strains circulating in hospital wastewaters indicated a warning danger for the transfer of these genes to the environment. Therefore, immediate measures are needed to control and treat hospital wastewaters more effectively.

## Ethical considerations

Ethical issues (Including plagiarism, informed consent, misconduct, data fabrication and/or falsification, double publication and/or submission, redundancy, etc.) have been considered by the authors.
